# Asymptomatic Bladder Metastasis from Breast Cancer

**DOI:** 10.1155/2014/672591

**Published:** 2014-03-04

**Authors:** Luigi Cormio, Francesca Sanguedolce, Giuseppe Di Fino, Paolo Massenio, Giuseppe Liuzzi, Nicola Ruocco, Pantaleo Bufo, Giuseppe Carrieri

**Affiliations:** ^1^Department of Urology and Renal Transplantation, University of Foggia, Viale Luigi Pinto 1, 71121 Foggia, Italy; ^2^Department of Pathology, University of Foggia, Viale Luigi Pinto 1, 71121 Foggia, Italy

## Abstract

*Introduction.* Breast cancer is the most common nondermatologic cancer in women. Common metastatic sites include lymph nodes, lung, liver, and bone. Metastases to the bladder are extremely rare, with all reported cases presenting with urinary symptoms. *Case Report.* Herein, we report the first case of completely asymptomatic bladder metastasis from breast cancer, occasionally revealed, 98 months after the initial diagnosis of lobular breast carcinoma, by a follow-up computed tomography scanning showing thickening of left bladder wall and grade II left hydronephrosis. A positive staining for estrogen and progesterone receptors was confirmed by immunohistochemistry. *Discussion.* The reported case confirms that bladder metastases from breast cancer tend to occur late after the diagnosis of the primary tumor and, for the first time, points out they can be asymptomatic. *Conclusion.* Such data support the need for careful follow-up and early intervention whenever such clinical situation is suspected.

## 1. Introduction

Breast cancer (BC) is the most common malignant disease affecting women, with the exception of nonmelanoma skin cancers, and the second leading cause of death for cancer in women, mainly due to metastatic spread [1, 2]. BC usually metastasizes to lung, bone, liver, lymph nodes, and skin but rarely spreads to other sites, such as urinary bladder and retroperitoneum [3]. As a matter of fact, there are less than 40 cases in the literature of bladder metastases from BC, with quite heterogeneous presentation and histological features. Herein, we report the first case of completely asymptomatic bladder metastasis from BC, occasionally revealed by a follow-up abdominal computed tomography (CT) scanning. Diagnostic and therapeutic challenges of such uncommon clinical condition are discussed.

## 2. Case Report

A 45-year-old female patient underwent right quadrantectomy with ipsilateral axillary node dissection followed by radical mastectomy in January 2005. Pathology revealed a pT2N3M0 lobular carcinoma, with positive estrogen receptor (ER, 70%) and progesterone receptor (PR, 80%) immunostainings, proliferative index (Ki67/MIB-1) as high as 15%, and negative HER2/neu status. The patient underwent 4 cycles of adjuvant chemotherapy with doxorubicin and cyclophosphamide, as well as locoregional radiotherapy. She also received hormonal treatment with LHRH analogues and tamoxifen until June 2007, when she was switched to exemestane. In January 2010, vertebral lumbar metastases were diagnosed and treated with radiotherapy, letrozole, and bisphosphonates. In October 2011, abdominal CT scan revealed para-aortic, interaortocaval, and iliac-obturator nodal metastases; therefore, further chemotherapy with capecitabine and vinorelbine was given.

**Figure 1:**
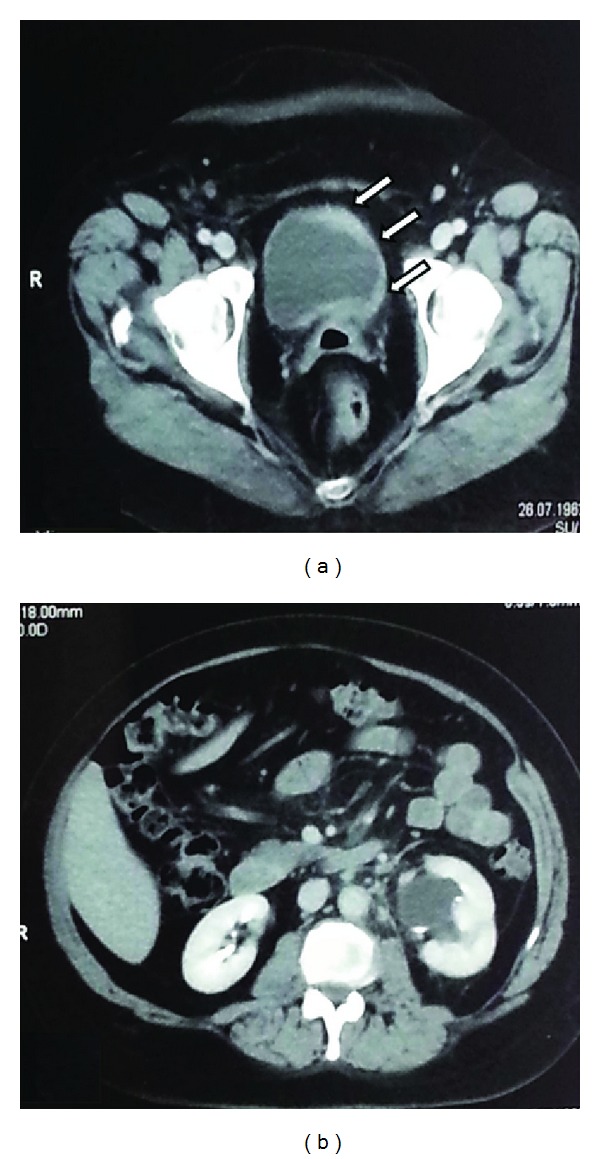
Abdominal computed tomography scanning showing (a) thickening of the left bladder wall (arrows) with grade II left hydronephrosis (b).

**Figure 2:**
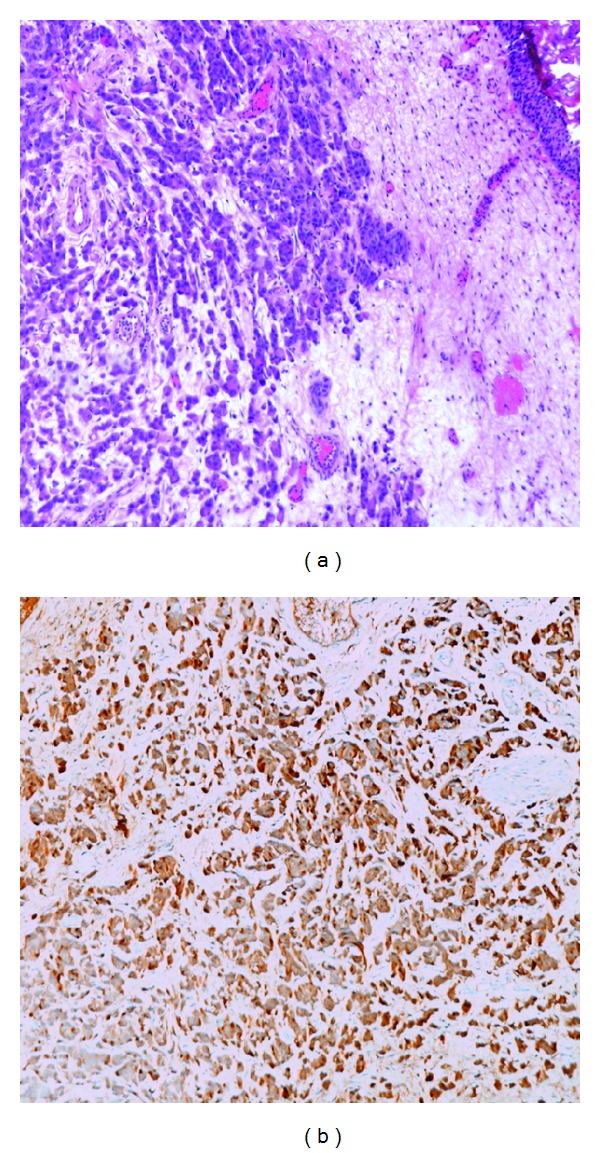
Metastatic lobular carcinoma. Bland-appearing tumor cells are arranged in strands with occasional single-signet ring-like cells, invading the stroma (hematoxylin & eosin, Nikon E1000, original magnification ×100) (a) and metastatic cells stain positively for apocrine marker GCDFP-15 (Nikon E1000, original magnification ×100) (b).

In March 2013, a follow-up abdominal CT scan displayed thickening of the left bladder wall with a grade II left hydronephrosis (Figure 1). The patient had no urinary symptoms; serum creatinine was 1.68 mg/dL and urinary cytology was negative. She was scheduled for cystoscopy, bladder biopsies, and left ureteral stenting. Cystoscopy showed a reddish bladder mucosa around the left ureteral orifice that seemed to be occluded by an external mass; deep transurethral resection (TUR) of the area was carried out to clear the occluded ureteral orifice from solid lardaceous tissue underneath the reddish urothelium. A left double-J stent was finally left indwelled. Pathology showed an undifferentiated adenocarcinoma deeply infiltrating the bladder wall, featuring strands of loosely cohesive medium and small size with occasional signet ring-like cells, covered by normal urothelium (Figure 2(a)). Tumor cells were positive for CK 7, CK 19, GCDFP-15 (Figure 2(b)), ER, and PR, while they were negative for CK 20 and E-cadherin, thus suggesting bladder metastasis from lobular carcinoma of the breast. Survey FISH resulted negative for *Her2/neu* gene amplification in 17q11.2-12.

Two weeks later, abdominal ultrasound scanning showed grade II right hydronephrosis; serum creatinine was 1.96 mg/dL. Suspecting an inflammatory right ureteral occlusion due to previous TUR, the patient underwent cystoscopy and right ureteral stenting; the right ureteral orifice appeared occluded by a solid lardaceous tissue underneath a normally looking urothelium. The ureteral orifice was deeply resected and exposed, and a double-J stent was placed. Pathology confirmed a bladder metastasis from lobular carcinoma of the breast. Thereafter, she was given chemotherapy with taxanes. In May 2013, however, she presented with acute renal failure (serum creatinine 6.1 mg/dL) due to bilateral obstructive hydronephrosis. Following placement of bilateral nephrostomy, serum creatinine returned to 1.5 mg/dL and she resumed treatment with taxanes, which is currently ongoing.

## 3. Discussion

Secondary tumors of the urinary bladder are rare, accounting for 2% of all bladder neoplasms [3]. The majority of them are due to direct extension of another pelvic neoplasm, such as prostate, large bowel, and cervical cancer [4]; the minority are metastases originating from lymphoma/leukemias or, less frequently, from other solid tumors such as breast, lung, and skin primaries. The differential diagnosis between a primary poorly differentiated or undifferentiated bladder cancer and a bladder metastasis from another primary carcinoma can be difficult and relies heavily on pathologic evaluation.

In the reported case, a positive staining for ER and PR allowed the differential diagnosis between primary bladder adenocarcinoma and bladder metastasis from breast cancer to be relatively simple. Nevertheless, not all cases reported in the literature showed the bladder metastasis having the same ER and PR status of the primary breast cancer. Variance in receptor status has so far been described in a few cases [5–7], with the main putative mechanisms being polyclonality of breast cancer cells and shifted expression due to endocrine therapy [7]. In such cases, apart from the few morphological tips suggested by Mostofi et al. [8], the differential diagnosis between primary and secondary bladder cancer relies on other immunohistochemical markers, particularly GCDFP-15, which has high specificity but low sensitivity.

The most interesting feature of the reported case, however, was the absence of urinary symptoms. To our knowledge, this is the first reported case of an asymptomatic bladder metastasis from breast cancer being diagnosed only on the basis of follow-up CT scan. It is likely that, in the absence of such imaging data, the patient would have developed renal failure further complicating her clinical status as well as her possibility of receiving further chemotherapy. Few cases of bladder metastasis from breast cancer leading to symptomatic hydronephrosis, renal failure, and death are described [1, 3, 5, 7, 9, 10].

Another interesting feature is the possibility of bladder metastases from breast cancer occurring as late as 30 years after the initial diagnosis of the primary tumor, with a mean time of 90 months having been calculated [5]. As a matter of fact, the reported case of bladder metastasis occurred 98 months after the initial diagnosis of breast cancer.

## 4. Conclusions

Bladder metastases from breast cancer are rare and their diagnosis relies on pathologic evaluation and immunohistochemical staining; while concordance in ER and PR status between primary and secondary tumor makes the diagnosis easy, discrepancy makes it more complicated. The reported case confirms that bladder metastases from breast cancer tend to occur late after the diagnosis of the primary tumor and, for the first time, points out that they can be asymptomatic. Taken together, such data support the need for careful follow-up and early intervention whenever such clinical situation is suspected.

## Abbreviations

BC: Breast cancer
CT: Computed tomography
ER: Estrogen receptor
PR: Progesterone receptor
TUR: Transurethral resection.
